# A nomogram for predicting the risk of bronchopulmonary dysplasia in preterm infants: a prospective multicenter study

**DOI:** 10.3389/fped.2026.1680824

**Published:** 2026-04-30

**Authors:** Yanping Guo, Hua Peng, Songzhou Xu, Zhangbin Yu, Xudong Yan, Ying Liu, Guobing Chen

**Affiliations:** 1 Department of Pediatrics, Peking University Shenzhen Hospital, Shenzhen, China; 2 Department of Neonatology, Shenzhen People’s Hospital (the Second Clinical Medical College, Jinan University, The First Afffliated Hospital, Southern University of Science and Technology), Shenzhen, China

**Keywords:** bronchopulmonary dysplasia, multicenter study, nomogram, predictive model, preterm infants

## Abstract

**Background:**

Bronchopulmonary dysplasia (BPD) is a common and serious complication in very preterm infants, with potential long-term adverse effects. This study aimed to analyze the perinatal and postnatal risk factors for BPD in preterm infants, and to develop and validate a risk model for early postnatal risk stratification based on information available within the first 7 days of life.

**Methods:**

Data on preterm infants with gestational age < 32 weeks were prospectively collected from 28 hospitals in Shenzhen from January 2022 to December 2023. Neonates admitted to the NICU of Peking University Shenzhen Hospital and Shenzhen People’s Hospital were designated as the external validation cohort, whereas those from the other 26 NICUs were randomly divided into training and internal validation cohorts in an 8:2 ratio. BPD was the primary outcome and was diagnosed based on the 2018 revised criteria. Predictive factors were identified from the training cohort using logistic regression, and a nomogram model was constructed. Model performance was evaluated in terms of discrimination, calibration, and potential clinical utility.

**Results:**

A total of 1,336 preterm infants were included, with 801 in the training cohort, 214 in the internal validation cohort, and 321 in the external validation cohort. Among them, 259 infants (19.39%) were diagnosed with BPD. The nomogram model was based on six predictive factors: gestational age, birth weight (per 100 g increase), occurrence of preterm premature rupture of membranes (PPROM), antenatal corticosteroid (ACS) administration, early onset sepsis, and invasive mechanical ventilation (IMV) within the first seven days of life. The area under the curve (AUC)s were 0.812, 0.783, and 0.810 in the training, internal validation, and external validation cohorts, respectively, indicating acceptable discrimination. Calibration showed reasonable agreement between predicted and observed outcomes. Decision curve analysis suggested potential net benefit across a range of threshold probabilities, although clinical interpretation remains exploratory.

**Conclusion:**

This nomogram provides early postnatal risk stratification of BPD based on routinely available clinical information within the first week of life and may help identify preterm infants at higher risk.

## 1 Introduction

Bronchopulmonary dysplasia (BPD) is a prevalent chronic lung disease in preterm infants and a major contributor to morbidity and mortality in this population (1–5). With advancements in medical care, the survival rate of very preterm infants has significantly improved, but this has been accompanied by a rising incidence of BPD. According to data from the National Institute of Child Health and Human Development (NICHD) in 2019, the incidence of BPD among preterm infants with gestational age (GA) of less than 32 weeks was as high as 71.1% (6). In a separate multicenter study conducted in China in 2020, the reported incidence of BPD was 72.2% among extremely preterm infants with gestational age of 22 to 28 weeks (7). Given that this study population was restricted to more immature infants, who generally have a higher baseline risk of BPD, these findings should not be interpreted as directly comparable. BPD was first described by Northway et al. in 1967 (8). The most widely used diagnostic criteria to date were introduced by the NICHD in 2001, which involve diagnosing BPD at 28 days of life and stratifying its severity at 36 weeks postmenstrual age (PMA), 56 days of life, or at discharge (9). However, with the rapid advancements and diversification in respiratory support technologies, these criteria have become increasingly insufficient to address clinical needs. In 2018, Higgins et al. proposed updated diagnostic criteria for BPD, standardizing the evaluation to 36 weeks PMA and incorporating the mode of ventilation and oxygen concentration to better categorize disease severity (10). These updated criteria have since been widely adopted for clinical diagnosis and epidemiological research.

BPD is currently understood to result from the combined influence of various prenatal, perinatal, and postnatal factors on immature lung development. While no effective treatment exists, prevention and early comprehensive management have become the main strategies, focusing on early postnatal risk stratification based on information available within the first 7 days of life. To date, several BPD predictive models have been reported both domestically and internationally (11–16). However, systematic reviews have highlighted several limitations of existing models, including limited external validation, heterogeneous outcome definitions, and potential overfitting. Moreover, most of these models concentrate on risk factors within the first 14 days of life and are primarily based on the 2001 NICHD diagnostic criteria. With the introduction of updated diagnostic criteria, the assessment of BPD has shifted at 36 weeks PMA, highlighting the need to revise the existing predictive model according to the new standard to ensure its applicability and accuracy. Furthermore, due to differences in ethnicity and healthcare systems, these models may not be entirely applicable to the Chinese population.

Therefore, we established a prospective multicenter birth cohort of very preterm infants (GA <32 weeks) in Shenzhen, China. The aim of this study was to identify risk factors associated with BPD and to develop and externally validate a nomogram-based prediction model based on the updated diagnostic criteria. This model is intended to support early postnatal risk stratification and assist in identifying preterm infants at higher risk for BPD, with the aim of providing risk estimates based on early postnatal information.

## 2 Materials and methods

### 2.1 Study design and participants

This multicenter study prospectively collected data from the Shenzhen Neonatal Data Network (SNDN), encompassing preterm infants with GA <32 weeks who received treatment in 28 tertiary neonatal intensive care units (NICUs) across Shenzhen between January 2022 and December 2023. All participating hospitals completed standardized training before the study commenced. Inclusion Criteria: (1) Preterm infants with GA < 32 weeks; (2) Hospitalized for at least 7 days (this criterion was applied because the model was constructed using variables available during the first 7 days of life). Exclusion Criteria:(1) Infants who died within the first 7 days of life (to ensure complete collection of early postnatal variables; this may introduce potential survivor bias); (2) Infants with chromosomal or genetic abnormalities, or multiple congenital anomalies, Including congenital lung diseases (e.g., congenital pulmonary hypoplasia, pulmonary sequestration, congenital bronchopulmonary cyst, transparent lung, or congenital pulmonary arteriovenous fistula), congenital heart diseases (e.g., atrial septal defect, ventricular septal defect, pulmonary valve stenosis, aortic valve stenosis, or Tetralogy of Fallot), or other systemic malformations. The dataset from Peking University Shenzhen Hospital and Shenzhen People’s Hospital were designated as the external validation set, while the datasets from the other centers were randomly allocated to the training and internal validation sets in an 8:2 ratio using computer-generated randomization. Center effects were considered but not modeled due to limited and imbalanced sample sizes across centers. The primary outcome was the development of BPD during hospitalization.

The design and reporting of this study adhered to the Transparent Reporting of a Multivariable Prediction Model for Individual Prognosis or Diagnosis (TRIPOD) guidelines (17). This study received approval from the Ethics Committee of Shenzhen People’s Hospital (Approval No. LL-KY-2022494-02), and other participating institutions agreed. The study protocol has been registered with the Chinese Clinical Trial Registry (ChiCTR2400090262).

### 2.2 Data collection and definitions

Data were retrieved from the electronic medical record systems of each participating institution using a standardized collection form. The following information was recorded:(1) Maternal information: age, ethnicity (Han Chinese or others), gestational hypertension, gestational diabetes, chorioamnionitis (defined based on placental histopathological diagnostic criteria after delivery) (18), occurrence of preterm premature rupture of membranes (PPROM) (defined as rupture of membranes lasting ≥18 h before delivery).(2) Perinatal information: antenatal corticosteroid (ACS) administration (defined as completion of a full course of antenatal corticosteroids), cervical cerclage, mode of conception (natural conception or assisted reproduction), multiple births (twins or more), mode of delivery (vaginal delivery or cesarean section), 1-minute Apgar score, 5-minute Apgar score. (3) Neonatal information: sex, GA, birth weight (BW), birth weight to gestational age ratio (RBG) {appropriate for gestational age(AGA), small for gestational age (SGA), large for gestational age (LGA)}, neonatal respiratory distress syndrome (RDS) (19), pulmonary surfactants (PS) usage, persistent pulmonary hypertension of the newborn (PPHN) (20), Mode of initial respiratory support (IRS) — the mode refers to the first type of respiratory support administered within 24 h after birth (According to the level of respiratory assistance, IRS was classified into three categories: oxygen therapy (supplemental oxygen without positive pressure support), non-invasive ventilation (including CPAP, BiPAP, or high-flow nasal cannula providing positive airway pressure without endotracheal intubation), and invasive mechanical ventilation (ventilatory support via endotracheal intubation using conventional or high-frequency ventilation modes). This variable was used to reflect the initial severity of respiratory compromise and early respiratory management strategy), invasive mechanical ventilation (IMV) within 7 days of birth (whether invasive mechanical ventilation was administered within the first 7 days after birth), early onset sepsis (EOS) (defined as infection within 72 h after birth, confirmed by clinical diagnosis or sterile body fluid culture) (21), and BPD. This model was designed to enable early postnatal risk stratification of BPD based on data collected within the first 7 days after birth; therefore, variables not available within this time frame were excluded. No missing data were observed for the variables included in the final analysis. Please see Supplementary Table 1 in the attached materials.

In this study, participants were categorized into the BPD group and the non-BPD group based on the presence of BPD at 36 weeks PMA. The diagnosis of BPD followed the 2018 revised criteria (10): preterm infants with GA ≤32 weeks, confirmed persistent pulmonary parenchymal disease on imaging, requiring respiratory support or supplemental oxygen for at least three consecutive days at 36 weeks PMA to maintain arterial oxygen saturation between 90% and 95%. Non-invasive respiratory support methods included continuous positive airway pressure (CPAP), non-invasive positive pressure ventilation, bi-level positive airway pressure, nasal high-flow oxygen therapy, and non-invasive high-frequency oscillatory ventilation. For infants who died after 7 days of life, BPD was assessed according to the standard evaluation protocol. For infants who did not reach 36 weeks PMA, BPD was determined based on oxygen requirements at discharge or follow-up data.

### 2.3 Sample size

The sample size for model development was determined based on the rule of having at least 10 events per predictor variable (22, 23). Accordingly, the number of BPD events in the training set was more than 10 times the number of predictive independent variables.

### 2.4 Statistical analysis

The normality of quantitative data was assessed using histograms and the Kolmogorov–Smirnov test. Normally distributed variables were expressed as mean ± standard deviation, whereas non-normally distributed variables were summarized as interquartile range. Categorical variables were presented as frequencies and percentages (%).

In the training set, univariate logistic regression was first performed on the collected feature data to identify predictors associated with BPD in preterm infants. All variables with clinical relevance or support from prior literature were included in the candidate pool, and selection was not solely based on univariable *P* values. Candidate variables were then refined to construct a multivariable logistic regression model using forward stepwise, backward stepwise, and bidirectional stepwise selection methods. No missing data were observed across all candidate variables; therefore, complete-case analysis was performed without imputation, ensuring consistency across model development and validation. The final nomogram was determined according to the Akaike Information Criterion (AIC), with the model presenting the lowest AIC value selected.

Model performance was evaluated and validated in the training, internal validation, and external validation sets. Receiver operating characteristic (ROC) curves were generated to assess the discriminative ability of the model, and the area under the curve (AUC) was calculated. An AUC > 0.7 was considered indicative of good discrimination (24). The Youden index, positive predictive value, and negative predictive value were calculated to further assess the model’s discriminative ability. Model calibration was qualitatively evaluated using a calibration plot, with predicted values closer to the diagonal line indicating better calibration. Quantitative assessment of calibration was performed using the Hosmer–Lemeshow goodness-of-fit test and the Brier score, with good calibration defined as *P* > 0.05 and Brier score < 0.25 (25). The clinical utility of the predictive model was assessed using decision curve analysis (DCA).

Statistical analyses were conducted using SPSS version 25.0 (IBM, Armonk, NY, USA), R version 4.4.1 (R Foundation for Statistical Computing, Vienna, Austria), and Stata version 18.0 (StataCorp, College Station, TX). A two-sided *P* value < 0.05 was considered statistically significant.

## 3 Results

### 3.1 General characteristics

**Figure 1:**
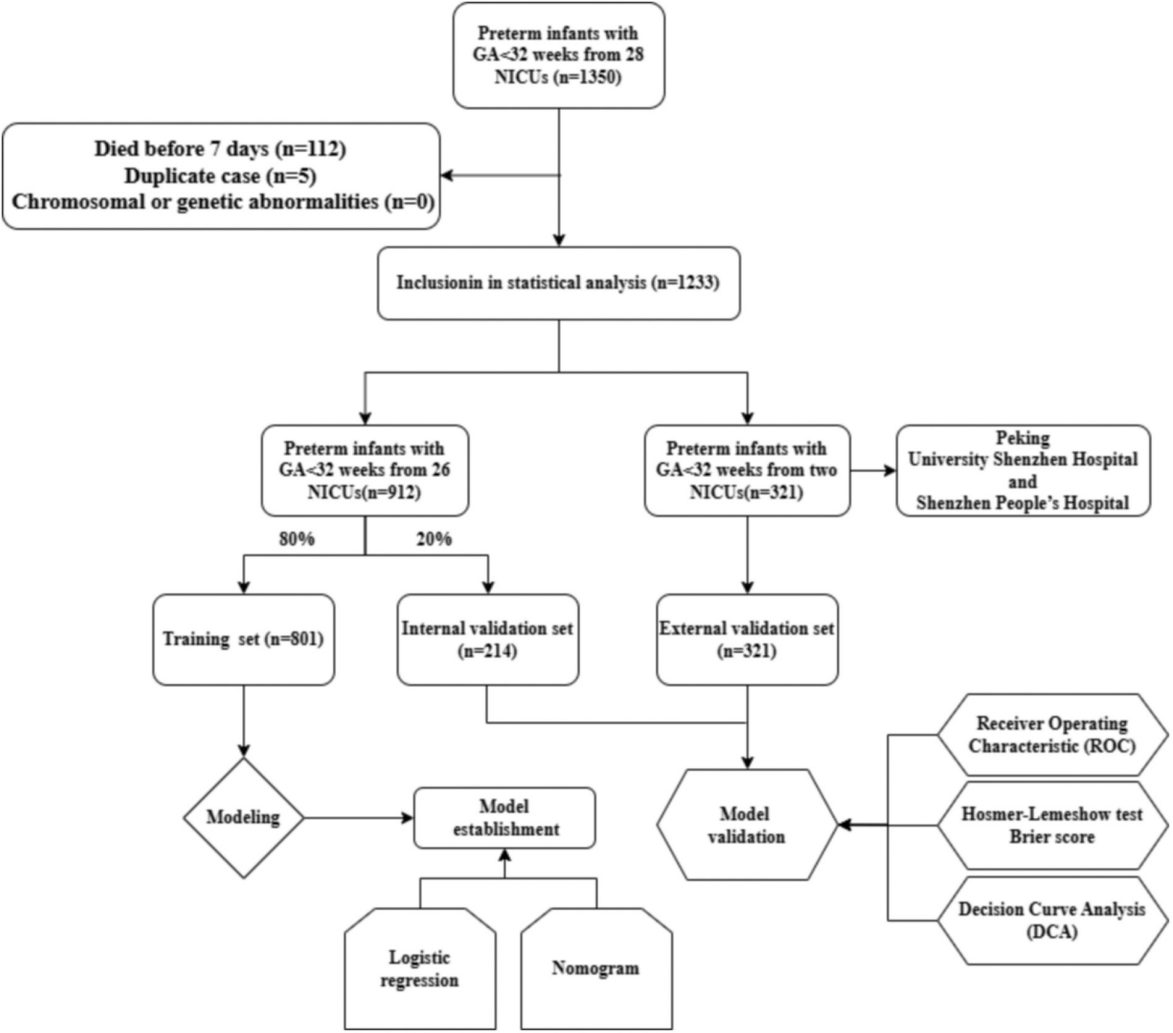
Flowchart depicting the study population and the development and validation of the nomogram prediction model.

The study included a training set of 801 participants and an internal validation set of 214 participants. In the training set, 152 cases of BPD (18.98%) were identified, whereas 41 cases of BPD (19.16%) were observed in the internal validation set. The external validation set comprised 321 participants, with 66 cases of BPD (20.56%). Figure 1 depicts the selection process of the study population and the development and validation of the nomogram prediction model. The demographic characteristics of the study population are summarized in Table 1.

**Table 1:** Baseline characteristics of All patients in the training and validation sets.

Variables	External validation set (*n* = 321)	Internal validation (*n* = 214)	Training set (*n* = 801)	Statistic	*P*
GA, weeks, M (Q₁, Q₃)	30.00 (28.57,31.14)	30.14 (28.43,31.14)	30.14 (28.57,31.14)	*χ*²=0.31#	0.855
BW, 100g, M (Q₁, Q₃)	12.70 (10.45,14.90)	13.00 (10.40,15.90)	13.00 (10.50,15.20)	χ²=2.56#	0.278
Monther Age, years, M (Q₁, Q₃)	32.00 (29.00,34.00)	32.00 (28.00,35.00)	31.00 (28.00,35.00)	χ²=0.71#	0.701
1-min Apgar score, M (Q₁, Q₃)	8.00 (8.00,9.00)	9.00 (8.00,10.00)	9.00 (8.00,10.00)	χ²=2.37#	0.305
5-min Apgar score, M (Q₁, Q₃)	10.00 (9.00,10.00)	10.00 (9.00,10.00)	10.00 (9.00,10.00)	χ²=4.89#	0.087
Sex, male, n(%)	140 (43.61)	91 (42.52)	333 (41.57)	χ²=0.40	0.818
Han Chinese, n(%)	312 (97.20)	208 (97.20)	771 (96.25)	χ²=0.87	0.646
Gestational hypertension, n(%)	54 (16.82)	45 (21.03)	141 (17.60)	χ²=1.72	0.424
Gestational diabetes, n(%)	81 (25.23)	59 (27.57)	222 (27.72)	χ²=0.74	0.690
Chorioamnionitis, n(%)	149 (46.42)	86 (40.19)	340 (42.45)	χ²=2.32	0.314
PPROM, n(%)	134 (41.74)	77 (35.98)	315 (39.33)	χ²=1.79	0.409
ACS, n(%)	283 (88.16)	196 (91.59)	724 (90.39)	χ²=1.94	0.378
Cerclage cervix, n(%)	26 (8.10)	14 (6.54)	71 (8.86)	χ²=1.22	0.544
assisted reproduction, n(%)	66 (20.56)	45 (21.03)	160 (19.98)	χ²=0.14	0.934
Multiple births, n(%)	72 (22.43)	62 (28.97)	232 (28.96)	χ²=5.24	0.073
Cesarean section, n(%)	217 (67.60)	142 (66.36)	549 (68.54)	χ²=0.40	0.821
SGA, n(%)	20 (6.23)	9 (4.21)	44 (5.49)	χ²=1.02	0.600
RBG, n(%)				χ²=1.98	0.739
AGA	282 (87.85)	193 (90.19)	720 (89.89)		
SGA	20 (6.23)	9 (4.21)	44 (5.49)		
LGA	19 (5.92)	12 (5.61)	37 (4.62)		
PS, n(%)	199 (61.99)	127 (59.35)	495 (61.80)	χ²=0.48	0.786
RDS, n(%)	264 (82.24)	175 (81.78)	654 (81.65)	χ²=0.05	0.973
EOS, n(%)	22 (6.85)	15 (7.01)	61 (7.62)	χ²=0.24	0.889
IMV, n(%)	160 (49.84)	109 (50.93)	398 (49.69)	χ²=0.11	0.948
IRS, n(%)				χ²=6.91	0.141
Oxygen therapy	11 (3.43)	2 (0.93)	11 (1.37)		
Non-invasive	175 (54.52)	119 (55.61)	458 (57.18)		
Invasive	135 (42.06)	93 (43.46)	332 (41.45)		
BPD2018, n(%)	66 (20.56)	41 (19.16)	152 (18.98)	χ²=0.38	0.828

#, kruskal-waills test; χ², chi-square test; M, median; Q₁, 1st quartile; Q₃, 3st quartile; PPROM, premature rupture of membranes; GA, gestational age; BW, birth weight; ACS, antenatal corticosteroid; RBG, birth weight to gestational age ratio; AGA, appropriate for gestational age; SGA, small for gestational age; LGA, large for gestational age; PS, pulmonary surfactants; RDS, neonatal respiratory distress syndrome; EOS, early onset sepsis; IRS, initial respiratory support (the mode refers to the first type of respiratory support administered within 24 h after birth, categorized as oxygen therapy, non-invasive ventilation, or invasive mechanical ventilation according to increasing support intensity); IMV, invasive mechanical ventilation within the first seven days of life; BPD, bronchopulmonary dysplasia. IRS refers to the initial respiratory support within 24 h after birth, whereas IMV refers to the use of invasive mechanical ventilation within the first 7 days of life; therefore, these two variables represent different time windows and clinical concepts and are not directly comparable.

### 3.2 Development of the nomogram prediction model

Univariate logistic regression analysis was performed on all variables in the training set, identifying univariate predictors associated with BPD in preterm infants (Table 2). Variables considered clinically relevant or supported by prior literature were selected as candidate predictors and entered into the multivariable logistic regression analysis, rather than being chosen solely based on univariable *P* values. The final candidate variables included GA, BW, PPROM, ACS, cervical cerclage, vaginal delivery, 1-minute Apgar score (AP1), 5-minute Apgar score (AP5), RDS, EOS, IMV, and IRS. All candidate variables were entered into multivariable logistic regression models, and multiple variable selection strategies were applied. The enter method was first used to construct full models including all candidate variables. In addition, stepwise logistic regression was performed using forward selection, backward elimination, and bidirectional approaches. Different entry (pe) and removal (pr) probability thresholds were specified (pe = 0.20, 0.01, 0.30, 0.05; pr = 0.02, 0.20) to evaluate the robustness of variable selection and model stability. AIC values were calculated for each model, and models generated by backward and bidirectional stepwise selection yielded identical results and achieved the lowest AIC values. Ultimately, a nomogram prediction model was developed based on the six variables corresponding to the lowest AIC: gestational age, birth weight, PPROM, antenatal ACS administration, EOS, and IMV within the first seven days of life. Table 3 summarizes the results of the multivariate logistic regression analysis. Given that the final model included six predictors, the training set required at least 60 BPD cases according to the events-per-variable principle. In this study, the training set contained 152 BPD cases, satisfying the sample size requirement.

**Table 2:** Univariate logistic regression analysis of predictors for BPD in preterm infants in the training set.

Variables	*β*	S.E	Z	*P*	OR (95%CI)
GA	−0.62	0.06	−10.60	<.001	0.54 (0.48∼0.60)
BW (per 100 g increase)	−0.34	0.04	−9.13	<.001	0.71 (0.66∼0.77)
Sex (female)	0.01	0.18	0.03	0.972	1.01 (0.70∼1.44)
Monther Age	−0.01	0.02	−0.35	0.723	0.99 (0.96∼1.03)
Gestational hypertension	−0.04	0.24	−0.18	0.858	0.96 (0.60∼1.53)
Gestational diabetes	0.04	0.20	0.18	0.861	1.04 (0.70∼1.53)
Chorioamnionitis	−0.05	0.18	−0.28	0.782	0.95 (0.66∼1.36)
PPROM	−0.38	0.19	−1.98	0.048	0.68 (0.47∼0.99)
ACS	−0.53	0.27	−1.93	0.053	0.59 (0.34∼1.01)
Cerclage cervix	0.88	0.27	3.26	0.001	2.40 (1.42∼4.07)
assisted reproduction	−0.07	0.23	−0.31	0.759	0.93 (0.60∼1.46)
Multiple births	−0.08	0.20	−0.40	0.688	0.92 (0.62∼1.37)
Vaginal delivery	0.44	0.19	2.35	0.019	1.55 (1.08∼2.24)
SGA	−0.89	0.53	−1.67	0.095	0.41 (0.14∼1.17)
1-min Apgar score	−0.17	0.04	−4.06	<.001	0.84 (0.78∼0.92)
5-min Apgar score	−0.21	0.07	−3.07	0.002	0.81 (0.71∼0.93)
PS	1.15	0.22	5.20	<.001	3.16 (2.05∼4.87)
RDS	0.77	0.28	2.72	0.007	2.15 (1.24∼3.73)
EOS	0.97	0.28	3.43	<.001	2.65 (1.52∼4.61)
IMV	1.80	0.22	8.05	<.001	6.06 (3.91∼9.40)

OR, odds ratio; CI, confidence interval; GA, gestational age; BW, birth weight; PPROM, premature rupture of membranes; ACS, antenatal corticosteroid; SGA, small for gestational age; PS, pulmonary surfactants; RDS, neonatal respiratory distress syndrome; EOS, early onset sepsis; IMV, invasive mechanical ventilation within the first seven days of life.

**Table 3:** Multivariate logistic regression analysis of the predictive factors.

Variables	β	S.E	Z	*P*	OR (95%CI)
Intercept	11.088	2.299	4.824	<.001	65410.004 (722.656∼5920478.875)
GA	−0.403	0.091	−4.445	<.001	0.668 (0.559∼0.798)
BW(per 100 g increase)	−0.086	0.054	−1.590	0.112	0.918 (0.825∼1.020)
PPROM	−0.346	0.218	−1.592	0.111	0.707 (0.462∼1.083)
ACS	−0.415	0.322	−1.289	0.198	0.661 (0.351∼1.241)
EOS	0.447	0.341	1.312	0.190	1.564 (0.802∼3.049)
IMV	1.131	0.244	4.641	<.001	3.100 (1.922∼4.998)

OR, odds ratio; CI, confidence interval; GA, gestational age; BW, birth weight; PPROM, premature rupture of membranes; ACS, antenatal corticosteroid; EOS, early onset sepsis; IMV, invasive mechanical ventilation within the first seven days of life

**Figure 2:**
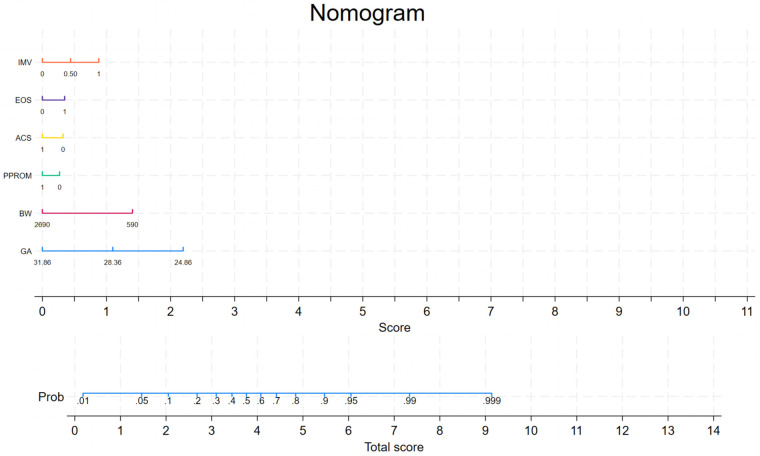
A nomogram model for predicting the occurrence of BPD during hospitalization among very preterm infants. GA, gestational age; BW, birth weight; PPROM, premature rupture of membranes; ACS, antenatal corticosteroid; EOS, early onset sepsis; IMV, invasive mechanical ventilation within the first seven days of life.

In the nomogram prediction model (Figure 2), each predictive variable was assigned a score for every case, and the sum of these scores yielded a total score. This total score was then used to estimate the probability of developing BPD during hospitalization in very preterm infants.

### 3.3 Model evaluation and validation

**Figure 3:**
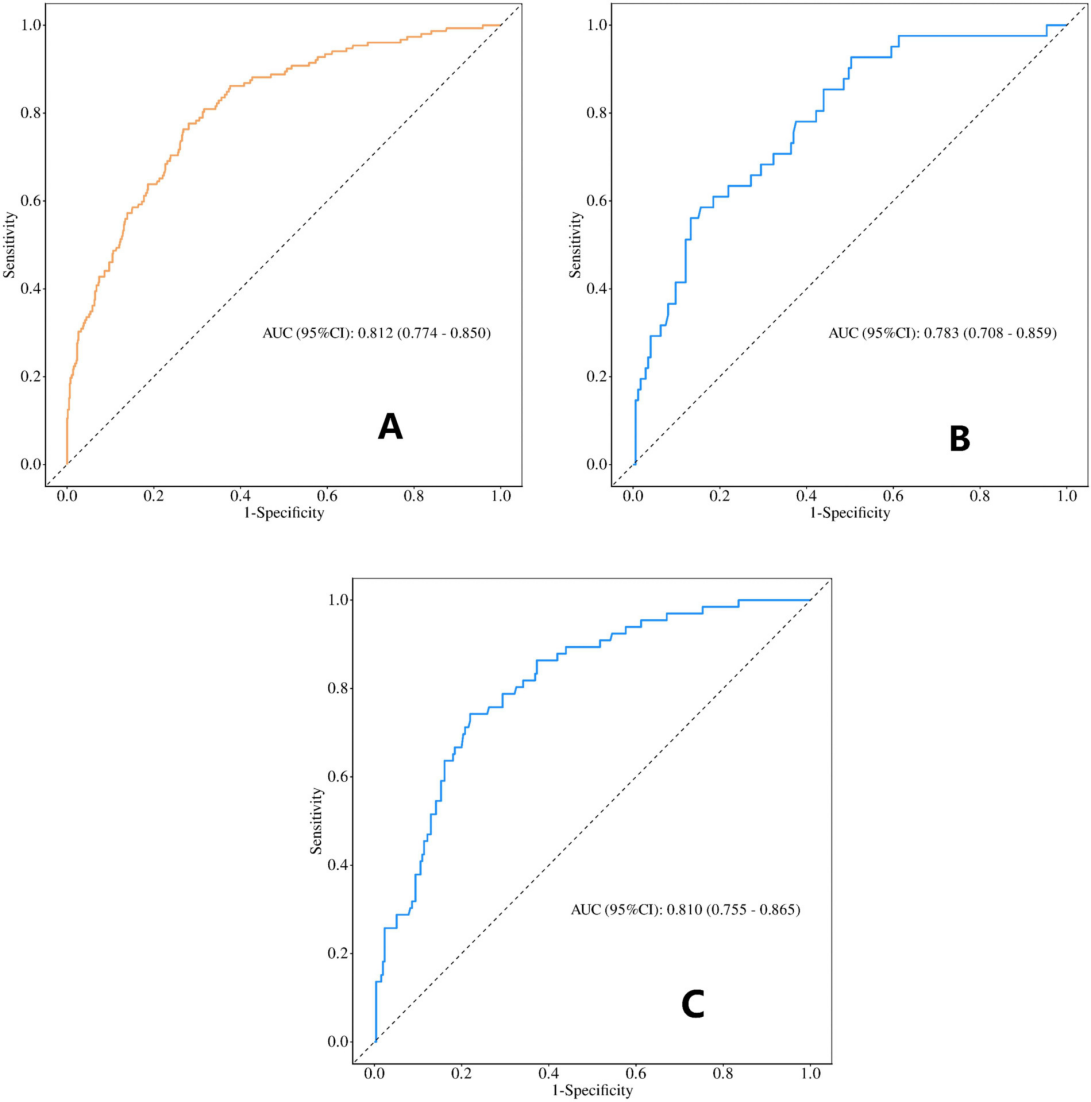
Validation of the prediction model using the receiver operating characteristic (ROC) curve. **(A)** Training set(*n*=801); **(B)** Internal validation set(*n*=214); **(C)** External validation set(*n*=321). (AUC, the area under the ROC curve.).

Roc curve analysis showed that the AUC was 0.812(95% CI 0.774-0.850) for the training set, 0.783(95% CI 0.708–0.859) for the internal validation set, and 0.810(95% CI 0.755–0.865) for the external validation set (Figure 3). All AUC values exceeded 0.75, suggesting satisfactory discriminative performance of the model. The positive predictive values for the training, internal validation, and external validation sets were 0.39, 0.35, and 0.41, respectively, while the corresponding negative predictive values were 0.93, 0.90, and 0.93. The cutoff value of 0.175 was determined in the training set using Youden’s index and applied unchanged to the internal and external validation cohorts.

**Figure 4:**
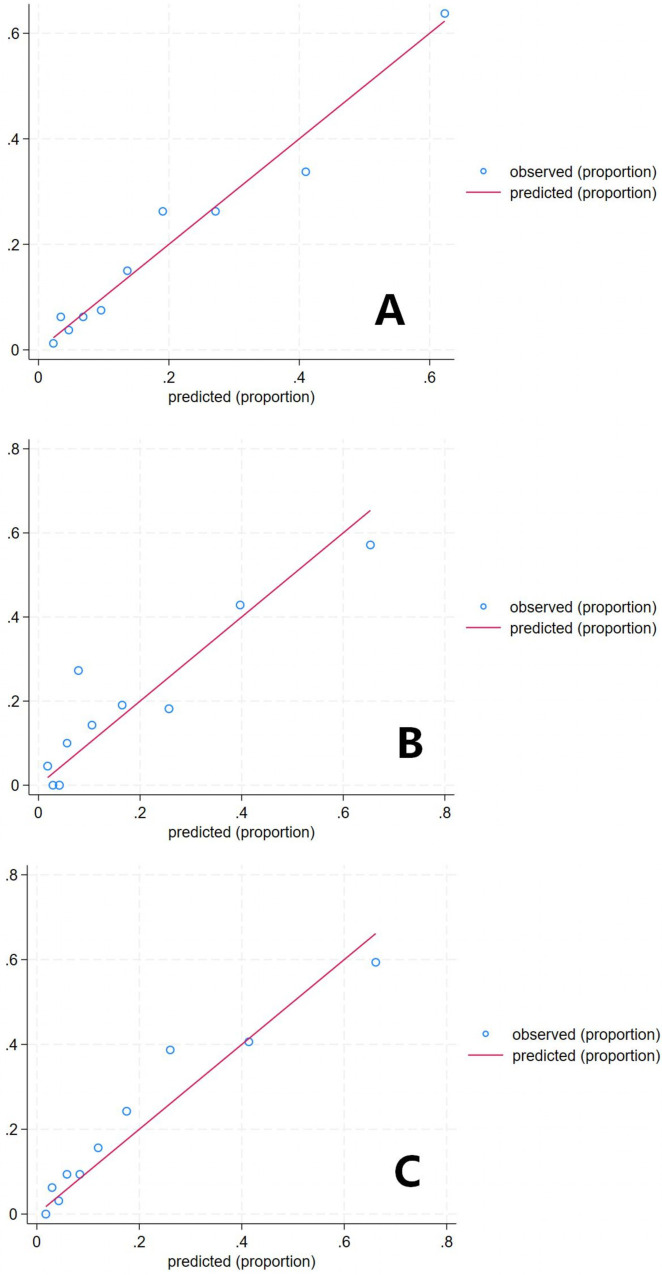
The calibration scatter plot of the predictive model. **(A)** Training set(*n*=801); **(B)** Internal validation set(*n*=214); **(C)** External validation set(*n*=321).

Regarding model calibration, the predicted values for the training, internal validation, and external validation sets closely aligned with the diagonal line (Figure 4). The Hosmer–Lemeshow goodness-of-fit test yielded *P*-values of 0.473, 0.461, and 0.611 for the respective sets, all exceeding 0.05. Likewise, the Brier scores were 0.118, 0.130, and 0.131, all below 0.25. These findings collectively suggest acceptable calibration, although they should be interpreted in conjunction with the limitations of each individual metric.

**Figure 5:**
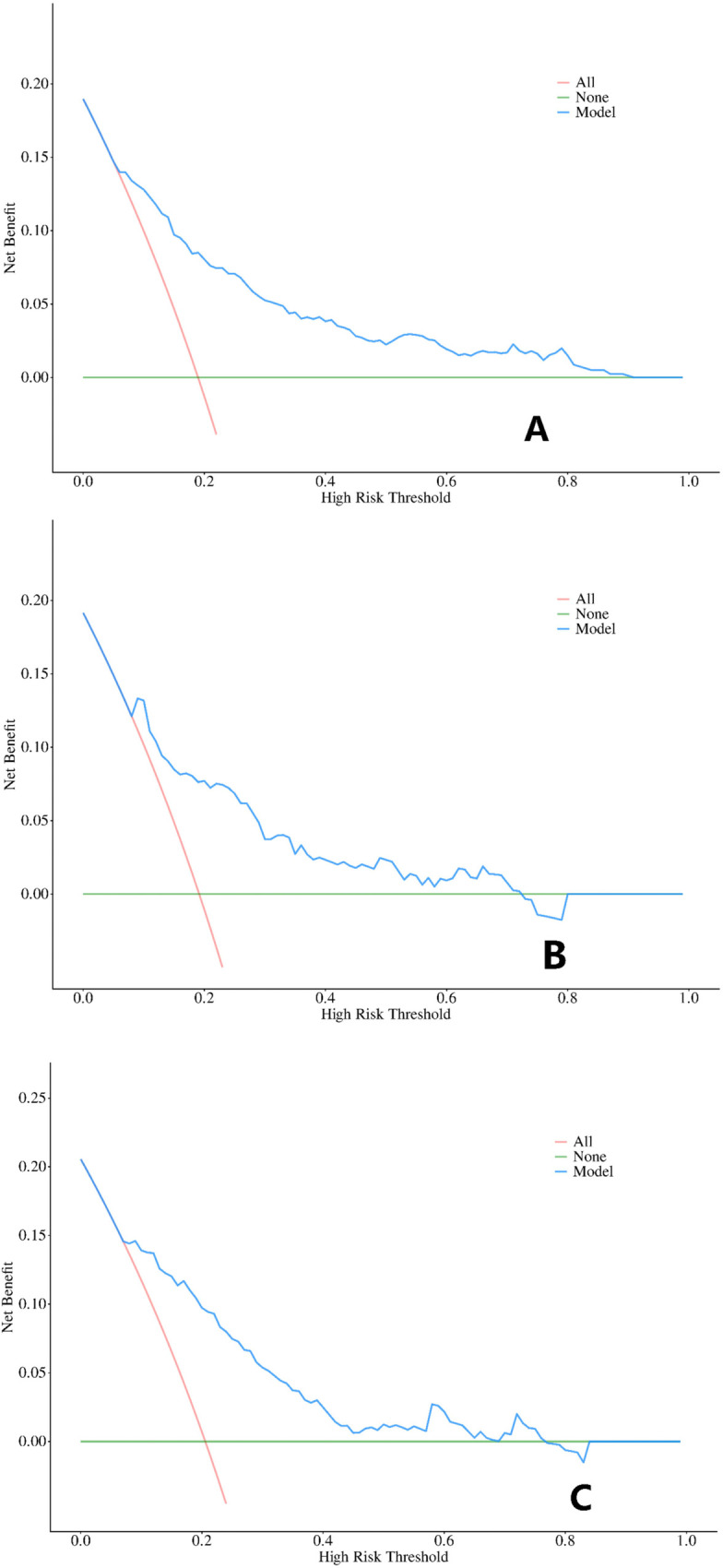
Decision curve analysis of the predictive model. **(A)** Training set(*n*=801); **(B)** Internal validation set(*n*=214); **(C)** External validation set(*n*=321).

Regarding clinical relevance, decision curve analysis (DCA) demonstrated a potential net benefit across a range of threshold probabilities in the training, internal validation, and external validation sets (Figure 5). However, the clinical interpretation of these threshold ranges remains exploratory, and the DCA results should be considered as supportive evidence of potential utility rather than definitive proof of clinical effectiveness.

## 4 Discussion

To our knowledge, this is among the few prospective multicenter studies in China involving very preterm infants that have developed a BPD prediction model using the updated 2018 NICHD criteria. The model aims to predict the risk of BPD in preterm infants with GA <32 weeks during hospitalization. It includes six predictive factors within the first week of life: gestational age, birth weight, PPROM, ACS administration, EOS, and IMV within the first seven days of life. These variables were selected based on a combination of clinical relevance and prior evidence, rather than solely on univariable statistical significance, to construct a prediction model for early postnatal risk stratification rather than causal inference.

It is important to note that while all six variables were included in the final model for prediction, GA and IMV were the strongest statistically supported contributors in the multivariable analysis, showing conventional significance. In contrast, BW, PPROM, ACS, and EOS were retained in the model to improve predictive accuracy but were not independently confirmed as statistically significant within this dataset. These variables were selected based on a combination of clinical relevance and prior evidence, rather than solely on univariable statistical significance. Accordingly, their interpretation should remain predictive rather than causal or mechanistic.

Existing studies have demonstrated an inverse relationship between the incidence of BPD and both birth weight and GA, with lower values for each associated with higher risk of developing BPD (26–28). Our study observed the same trend. In the nomogram, predicted risk increases as birth weight and gestational age decrease, with the highest scores observed at a birth weight of 590 g and a GA of 24 + 6 weeks. These infants are at a critical stage of lung development, transitioning from the canalicular to the saccular phase, and premature birth results in structurally and functionally immature lungs (28). In addition, postnatal interventions such as mechanical ventilation and oxygen exposure may further disrupt lung development (29, 30). Taken together, these findings suggest that GA is a key contributor to BPD risk, while BW contributes to prediction but did not demonstrate independent statistical significance in the multivariable model and should therefore be interpreted cautiously.

In this study, PPROM appeared to have a seemingly protective effect on the development of BPD, which contrasts with typical clinical observations. Evidence from multivariable analyses suggests that the primary determinants of BPD are delivery before 24 weeks of gestation and prolonged oligohydramnios, rather than PPROM itself (31). Conversely, PROM, including PPROM, has also been associated with increased inflammatory exposure and a higher risk of BPD (32). These findings indicate a complex relationship. In our model, PPROM was retained as a predictive variable, but its independent association with BPD was not statistically confirmed and should not be interpreted causally.

ACS administration has been reported in prior studies as a potential protective factor against BPD, likely through promoting fetal lung maturation and reducing the need for respiratory support after birth (33, 34). However, in the present study, ACS was included as a predictive factor, and its independent effect was not statistically significant in multivariable analysis; therefore, it should be interpreted as a model component rather than an independent protective determinant.

EOS has also been associated with an increased risk of BPD in previous studies, possibly through inflammation-mediated pathways involving cytokine release and impaired alveolar development (35). However, in our model, EOS was retained for predictive purposes, and its independent association with BPD was not statistically confirmed after adjustment.

IMV within the first week of life was included in the predictive model. Previous studies have consistently shown that IMV contributes to lung injury through barotrauma, volutrauma, and oxygen toxicity (12, 30, 36). In our study, IMV remained one of the strongest predictors in the multivariable analysis. These findings underscore the importance in early postnatal risk stratification, rather than implying a causal interpretation alone.

This study diagnosed BPD based on the 2018 criteria and developed a predictive model using perinatal information and early data collected within the first 7 days after birth for preterm infants with GA <32 weeks. A nomogram was constructed and externally validated in an independent cohort, demonstrating moderate discrimination. Model performance was supported by ROC analysis, the Hosmer–Lemeshow test, decision curve analysis, and calibration plots, which indicated general agreement between predicted and observed outcomes. These results suggest that the model may be useful for early postnatal risk stratification. However, given the moderate predictive performance and inherent limitations of calibration and decision curve analyses, these findings should be interpreted as exploratory and supportive evidence rather than confirmatory support for clinical application. Accordingly, the model should be considered an exploratory prediction tool rather than a clinically implementable decision instrument, and further external validation is required before clinical use.

However, this study has several limitations. First, due to data constraints, some potential predictive factors (such as C-reactive protein, procalcitonin, or umbilical cord blood gases) were not included in the analysis. Second, all participating centers were located in Shenzhen, which may limit geographic diversity and generalizability. Third, although this was a multicenter study involving 28 NICUs, the sample size within each center was limited and the distribution of cases across centers was highly imbalanced. Therefore, center-level effects were not explicitly modeled in the current analysis. This may have influenced the stability and transportability of the model and may limit its generalizability across different clinical settings. Given the known heterogeneity in clinical practices, patient management strategies, and resource availability across neonatal intensive care units, the absence of explicit adjustment for center effects represents an important limitation. Future studies with larger and more balanced multicenter datasets should consider hierarchical or mixed-effects modeling approaches to better account for between-center variability and to further improve model robustness and external validity. In addition, to ensure the completeness of early variable data and to allow infants to reach 36 weeks of PMA for BPD outcome assessment, this study included only those with a hospital stay of ≥7 days and excluded infants who died within the first 7 days of life. While this approach is consistent with prior studies, it may introduce survivor bias by excluding infants with early mortality who may have had a different risk profile. This could potentially lead to underestimation or distortion of the true associations between predictors and BPD, and should be carefully considered when interpreting the findings. Future studies should incorporate data from a broader range of regions and institutions to further assess the robustness and generalizability of the model through external validation.

## 5 Conclusions

In conclusion, this study developed and externally validated a nomogram for early postnatal risk stratification of BPD in preterm infants based on routinely available clinical variables. The model provides individualized estimates of BPD risk and may help identify infants at elevated risk who may require closer monitoring and further clinical evaluation. However, its current role should be limited to risk prediction rather than clinical decision-making or implementation guidance.

## Data Availability

The original contributions presented in the study are included in the article/Supplementary Material, further inquiries can be directed to the corresponding authors.
