# Supplementary material for: A nomogram for predicting the risk of bronchopulmonary dysplasia in preterm infants: a prospective multicenter study
Source: Front Pediatr. 2026 Apr 30;14:1680824. doi: 10.3389/fped.2026.1680824 (PMC13171793; doi:10.3389/fped.2026.1680824)

In this study, case report forms (CRFs) were used to collect clinical data of enrolled patients through an online database, ensuring timely data entry and efficient quality control. The online database is available at [www.perinatalcloud.com](http://www.perinatalcloud.com) (Perinatal Cloud, Neonatal Data Collaboration Network), with the website and login interface shown in the figure below (data display from our center). This database has been used in several published studies, for example: Li T, Zhang G, Li R, et al. Survival and morbidity in very preterm infants in Shenzhen: a multi-center study. Front Pediatr. 2024;11:1298173. doi:10.3389/fped.2023.1298173.

This cohort is a multicenter prospective study. All cases from participating centers were entered through the “Perinatal Cloud” database, and all predefined variables were completed before patient discharge, resulting in essentially no missing data.
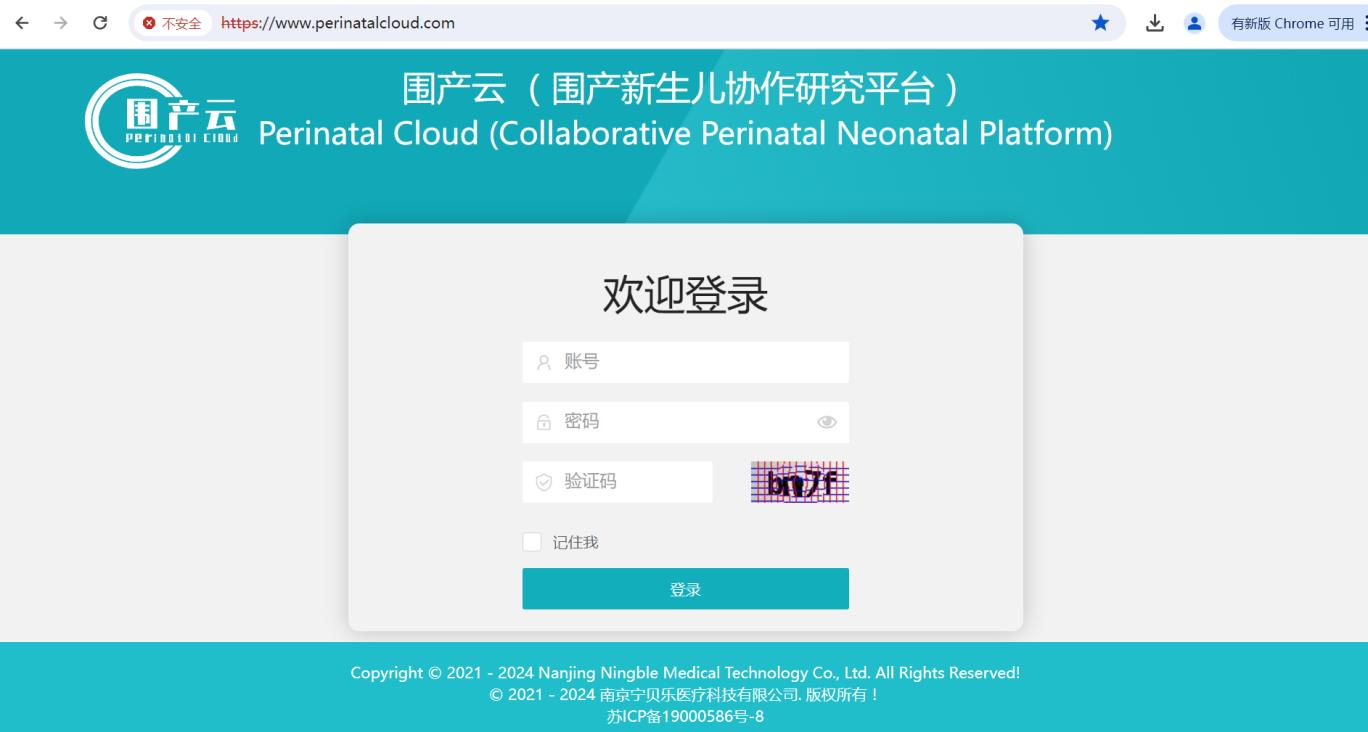


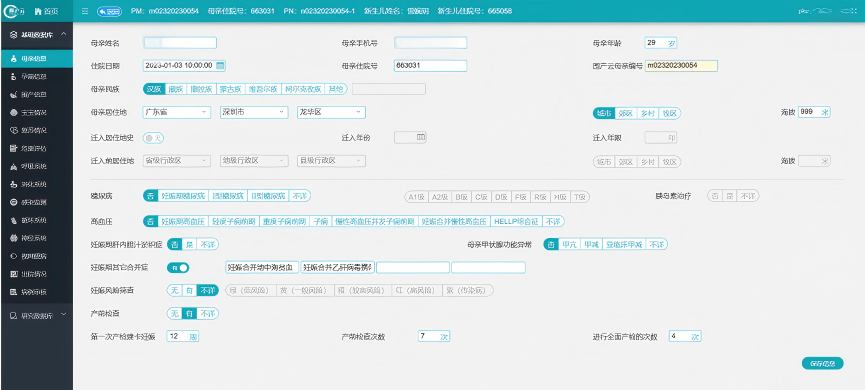


**Supplementary description:**

Based on the results of Table 2, variables were initially selected according to clinical relevance and evidence from previous literature. Although ACS did not reach statistical significance in univariate analysis (P = 0.053), it was retained as a candidate predictor due to its clinical importance and potential contribution to model performance, as supported by previous studies (Reference 33).

Univariate logistic regression analysis was performed on all variables in the training set, identifying univariate predictors associated with BPD in preterm infants (Table 2). Variables with P < 0.05 in the univariate analysis, as well as those considered clinically significant, were subsequently entered as independent variables into a multivariate logistic regression model.

The final candidate variables included GA, BW, PPROM, ACS, cervical cerclage, vaginal delivery, 1-minute Apgar score (AP1), 5-minute Apgar score (AP5), RDS, EOS, IMV, and IRS.

These candidate variables were entered into multivariable logistic regression models, and multiple variable selection strategies were applied to construct predictive models. First, the enter method was used to include all candidate variables simultaneously, generating several full models (enter, enter1, enter2, and enter3). Stepwise removal of variables was performed to evaluate model stability and consistency. In addition, stepwise logistic regression was conducted for variable selection. Different entry probabilities (pe) and removal probabilities (pr) were specified to construct multiple candidate models, including forward selection, backward elimination, and bidirectional stepwise selection. Different combinations of parameters were applied (pe = 0.20, 0.01, 0.30, 0.05; pr = 0.02, 0.20) to assess the impact of variable selection criteria on model stability and variable inclusion.

All models were stored using the Stata estimates store command, and model fit was compared using estimates stats. Finally, the Akaike Information Criterion (AIC) was used for model selection, and the model with the lowest AIC was identified as the optimal predictive model. The variables included in this final model correspond to the independent predictors reported in Table 3.

Akaike's information criterion and Bayesian information criterion


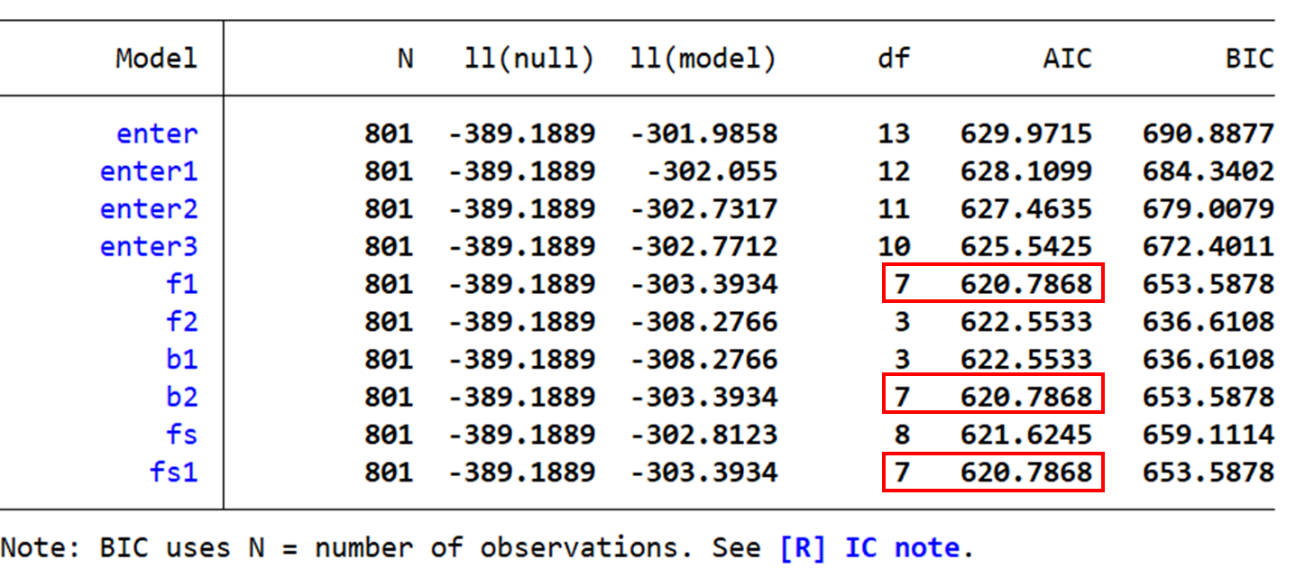


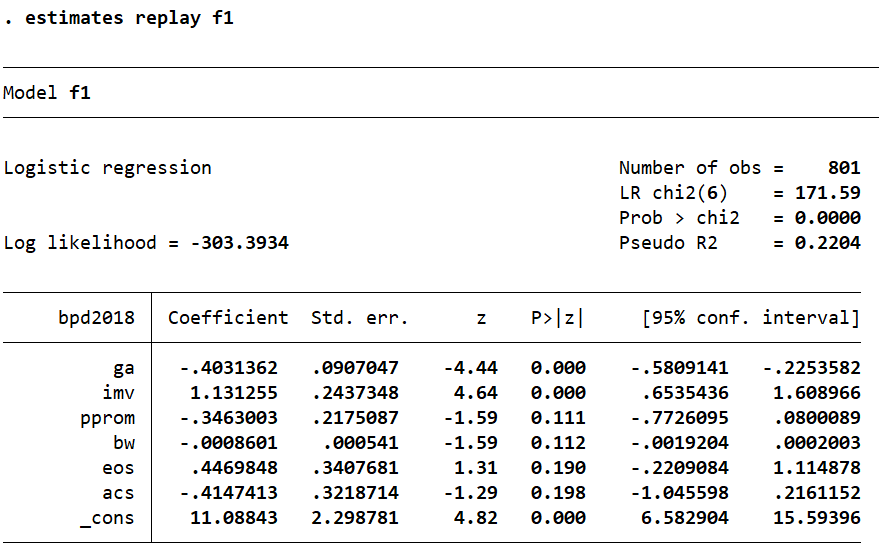


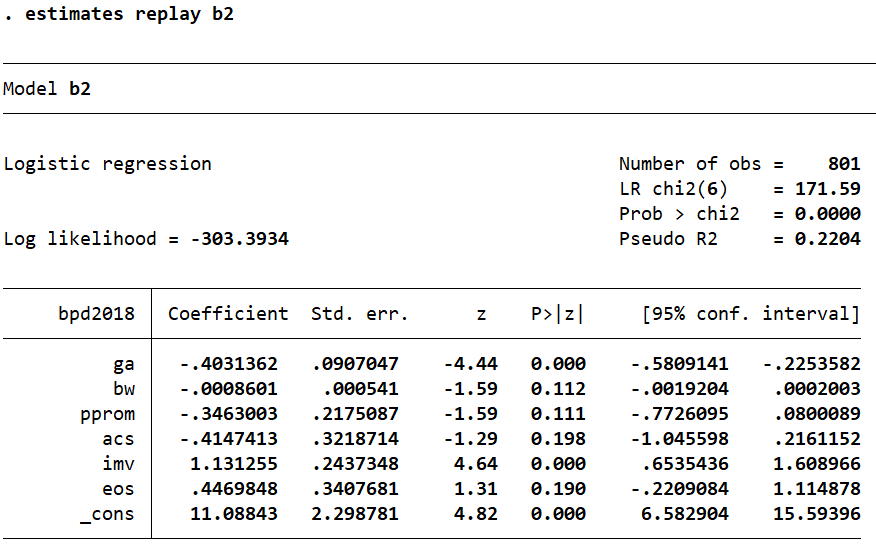


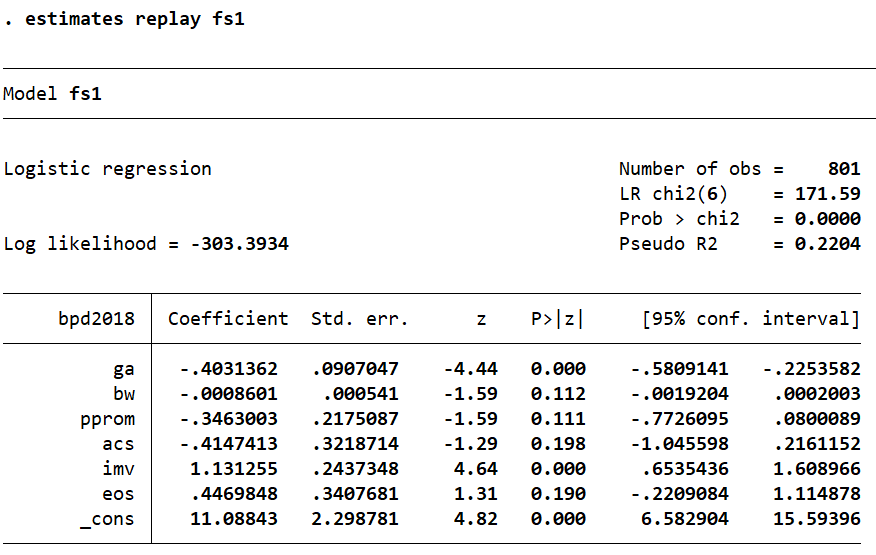

Supplement: Supplementary file 1 [file Supplementaryfile1.docx]
