# Supplementary material for: A nomogram for predicting the risk of bronchopulmonary dysplasia in preterm infants: a prospective multicenter study
Source: Front Pediatr. 2026 Apr 30;14:1680824. doi: 10.3389/fped.2026.1680824 (PMC13171793; doi:10.3389/fped.2026.1680824)
Supplement: Supplementary file 2 [file Table1.docx]

| Hospital ID | Cases (n) |
| --- | --- |
| 1 | 218 |
| 2 | 200 |
| 10 | 55 |
| 11 | 65 |
| 14 | 148 |
| 15 | 15 |
| 16 | 22 |
| 17 | 28 |
| 18 | 71 |
| 20 | 21 |
| 21 | 12 |
| 22 | 44 |
| 23 | 103 |
| 24 | 4 |
| 26 | 10 |
| 28 | 3 |
| 31 | 13 |
| 32 | 70 |
| 33 | 12 |
| 34 | 28 |
| 35 | 22 |
| 37 | 4 |
| 38 | 17 |
| 39 | 1 |
| 40 | 39 |
| 48 | 88 |
| 49 | 8 |
| 50 | 15 |

Table S1. Number of cases by hospital (numbers on the left represent hospitals).
