# Supplementary material for: A nomogram for predicting the risk of bronchopulmonary dysplasia in preterm infants: a prospective multicenter study
Source: Front Pediatr. 2026 Apr 30;14:1680824. doi: 10.3389/fped.2026.1680824 (PMC13171793; doi:10.3389/fped.2026.1680824)
Supplement: Supplementary file 3 [file Table2.docx]

Supplementary Table 2. Definitions, data sources, and timing of assessment of study variables

| **Variable** | **Definition** | **Data source** | **Timing of assessment** |
| --- | --- | --- | --- |
| Gestational age (GA) | Best obstetric estimate based on last menstrual period and/or early ultrasound | Electronic medical record (EMR), obstetric record | At birth |
| Birth weight (BW) | Measured within 1 hour after birth using calibrated scale | Neonatal medical record | At birth |
| Preterm premature rupture of membranes (PPROM) | Rupture of membranes occurring ≥18 hours before delivery | Obstetric record | Before delivery |
| Antenatal corticosteroids (ACS) | Completion of a full course of antenatal corticosteroid therapy prior to delivery | Obstetric medication record | Prenatal period |
| Cervical cerclage | Surgical placement of cervical suture during pregnancy | Obstetric record | Prenatal period |
| Mode of conception | Natural conception or assisted reproductive technology (ART) | Obstetric record | At enrollment |
| Multiple gestation | Twin or higher-order pregnancy | Obstetric record | Prenatal period |
| Mode of delivery | Vaginal delivery or cesarean section | Delivery record | At birth |
| 1-minute Apgar score (AP1) | Standard Apgar score assessed at 1 minute after birth | Delivery record | 1 minute after birth |
| 5-minute Apgar score (AP5) | Standard Apgar score assessed at 5 minutes after birth | Delivery record | 5 minutes after birth |
| Sex | Male or female | Neonatal record | At birth |
| Birth weight to gestational age ratio (RBG) | Classified as AGA, SGA, or LGA based on population-specific growth standards | Neonatal record | At birth |
| Respiratory distress syndrome (RDS) | Clinical diagnosis based on respiratory distress and radiographic findings | Neonatal record | ≤24 hours after birth |
| Pulmonary surfactant (PS) use | Administration of exogenous surfactant therapy | NICU record | Early neonatal period |
| Persistent pulmonary hypertension of the newborn (PPHN) | Diagnosed based on echocardiography and clinical criteria | NICU record | Neonatal period |
| Initial respiratory support (IRS) | First respiratory support mode within 24 hours after birth, categorized as oxygen therapy, non-invasive ventilation, or invasive mechanical ventilation | NICU record | ≤24 hours after birth |
| Invasive mechanical ventilation (IMV) | Use of invasive mechanical ventilation via endotracheal intubation within the first 7 days of life | NICU record | ≤7 days after birth |
| Early-onset sepsis (EOS) | Infection occurring within 72 hours after birth, diagnosed clinically and/or confirmed by sterile body fluid culture | NICU record | ≤72 hours after birth |
| Chorioamnionitis | Placental histopathological diagnosis of intrauterine infection/inflammation | Pathology report | Postnatal (after delivery) |
| Bronchopulmonary dysplasia (BPD) | Diagnosed according to NICHD 2018 criteria | Neonatal record / discharge diagnosis | At 36 weeks postmenstrual age or discharge |
